# Risk of cognitive decline among patients with dengue virus infection: a systematic review

**DOI:** 10.1093/ijnp/pyae053

**Published:** 2024-11-02

**Authors:** Lakshmi Thangavelu, Siddig Ibrahim Abdelwahab, Abdullah Farasani, Suhas Ballal, Pooja Bansal, Deepak Nathiya, Kiranjeet Kaur, M Ravi Kumar, Aashna Sinha, Hayam A Alrasheed, Maha F Al-Subaie, Nawal A Al Kaabi, Ali Al bshabshe, Mona A Al Fares, Hawra Albayat, Ali A Rabaan, Kumud Pant, Quazi Syed Zahiruddin, Arathi P Rao, Mahalaqua Nazli Khatib, Hassan Ahmad Alfaifi, Syam Mohan, Sanjit Sah, Prakasini Satapathy

**Affiliations:** Center for Global Health Research, Saveetha Medical College and Hospitals, Saveetha Institute of Medical and Technical Sciences, Saveeth University, Chennai 602105, India; Medical Research Centre, Jazan University, Jazan 45142, Saudi Arabia; Department of Medical Laboratory Technology, Faculty of Applied Medical Sciences, Jazan University, Jazan 45142, Saudi Arabia; Department of Chemistry and Biochemistry, School of Sciences, JAIN (Deemed to be University), Bangalore, Karnataka 560069, India; Department of Allied Healthcare and Sciences, Vivekananda Global University, Jaipur, Rajasthan 303012, India; Department of Pharmacy Practice, Institute of Pharmacy, NIMS University, Jaipur, Rajasthan 303121, India; Chandigarh Pharmacy College, Chandigarh Group of College, Jhanjeri, Mohali - 140307, Punjab, India; Department of Chemistry, Raghu Engineering College, Visakhapatnam, Andhra Pradesh 531162, India; School of Applied and Life Sciences, Division of Research and Innovation, Uttaranchal University, Dehradun 248007, India; Department of Pharmacy Practice, College of Pharmacy, Princess Nourah bint Abdulrahman University, Riyadh 11671, Saudi Arabia; Research Center, Dr. Sulaiman Alhabib Medical Group, Riyadh 13328, Saudi Arabia; College of Medicine, Alfaisal University, Riyadh 11533, Saudi Arabia; College of Medicine and Health Science, Khalifa University, Abu Dhabi, 127788, United Arab Emirates; Sheikh Khalifa Medical City, Abu Dhabi Health Services Company (SEHA), Abu Dhabi, 51900, United Arab Emirates; Adult Critical Care Department of Medicine, Division of Adult Critical Care, College of Medicine, King Khalid University, Abha 62561, Saudi Arabia; Department of Internal Medicine, King Abdulaziz University Hospital, Jeddah 21589, Saudi Arabia; Infectious Disease Department, King Saud Medical City, Riyadh7790, Saudi Arabia; Molecular Diagnostic Laboratory, Johns Hopkins Aramco Healthcare, Dhahran 31311, Saudi Arabia; Department of Public Health and Nutrition, The University of Haripur, Haripur 22610, Pakistan; Research Center, Dr. Sulaiman Alhabib Medical Group, Riyadh 13328, Saudi Arabia; Department of Biotechnology, Graphic Era (Deemed to be University), Clement Town, Dehradun 248002, India; Department of Allied Sciences, Graphic Era Hill University, Clement Town, Dehradun 248002, India; Global South Asia Infant Feeding Research Network (SAIFRN), Division of Evidence Synthesis, Global Consortium of Public Health and Research, Datta Meghe Institute of Higher Education, Wardha 442107, India; Department of Health Policy, Prasanna School of Public Health, Manipal Academy of Higher Education, Manipal 576104, India; Division of Evidence Synthesis, Global Consortium of Public Health and Research, Datta Meghe Institute of Higher Education, Wardha 442107, India; Pharmaceutical Care Administration (Jeddah Second Health Cluster), Ministry of Health, Jeddah, 22233, Saudi Arabia; Substance Abuse and Toxicology Research Centre, Jazan University, Jazan 45142, Saudi Arabia; School of Health Sciences, University of Petroleum and Energy Studies, Dehradun, Uttarakhand 248007, India; SR Sanjeevani Hospital, Kalyanpur, Siraha 56517, Nepal; Department of Paediatrics, Dr. D. Y. Patil Medical College, Hospital and Research Centre, Dr. D. Y. Patil Vidyapeeth, Pune 411018, Maharashtra, India; Department of Public Health Dentistry, Dr. D.Y. Patil Dental College and Hospital, Dr. D.Y. Patil Vidyapeeth, Pune 411018, Maharashtra, India; University Center for Research and Development, Chandigarh University, Mohali, Punjab 140413, India; Medical Laboratories Techniques Department, AL-Mustaqbal University, 51001 Hillah, Babil, Iraq

**Keywords:** dengue, cognitive impairment, dementia, Alzheimer, systematic review

## Abstract

Dengue fever, caused by the dengue virus and transmitted through Aedes mosquitoes, is a growing public health concern, particularly in tropical and subtropical regions. Traditionally associated with febrile and hemorrhagic symptoms, recent research suggests a potential link between dengue and cognitive impairments. This systematic review assessed existing research to understand the association between dengue virus infection and cognitive impairments, including dementia, Alzheimer disease, memory loss, and confusion. This systematic review followed preferred reporting items for systematic reviews and meta-analyses guidelines. A comprehensive literature search was conducted in PubMed, EMBASE, and Web of Science up to January 18, 2024. Studies examining the prevalence and association of cognitive impairments in dengue patients were included. Data extraction and quality assessment were performed using Nested Knowledge software and the Newcastle-Ottawa Scale. Of the 1129 articles identified, 5 were included in the review, covering a total of 200 873 participants from Taiwan, Brazil, and France. Evidence from population-based cohort studies indicated short-term cognitive impairments, including confusion and memory loss, in some dengue patients. Additionally, long-term risks of dementia, including Alzheimer disease and vascular dementia, were observed, particularly among older adults. Although the findings suggest there might be an association between dengue infection and cognitive decline, the mechanisms underlying this link remain unclear. This systematic review suggests that dengue virus infection may affect cognitive function in both acute and long-term contexts. However, the current evidence is not strong enough to establish a conclusive link. Further research with larger sample sizes and longitudinal studies is essential to confirm the impact of dengue virus on cognitive health.

## INTRODUCTION

Dengue fever, caused by the dengue virus and transmitted through Aedes mosquitoes, has become a notable public health concern worldwide, particularly in tropical and subtropical regions, affecting millions annually.^1^ Dengue manifests with symptoms ranging from mild to severe, significantly impacting global health. In 2019, there were 56.7 million new dengue cases globally, with a worldwide death toll of 36 055. From 1990 to 2019, the incidence of dengue rose steadily by 1.2% per year, a trend attributable to factors such as increased urbanization, global travel, and climate change, which facilitate the proliferation of Aedes mosquitoes, the primary vectors of the dengue virus.^2^

While the typical presentation of dengue includes fever, rash, and joint pain, recent research has uncovered a potential link between dengue virus infection and cognitive impairment, suggesting broader implications for individuals affected by the virus. In 2009, the WHO included CNS involvement in the definition of severe dengue.^3^ Neurological manifestations are now more frequently recognized, with the incidence of encephalopathy and encephalitis estimated at 0.5% to 6.2%.^4–6^ These complications, including metabolic disturbances and autoimmune reactions, can lead to serious long-term brain issues such as dementia and Alzheimer disease. However, the full extent and nature of these long-term consequences remain underexplored in the current scientific literature. There have also been observations of diminished sensitivity, cognitive impairment, convulsions, and a decline in cognitive function during acute phases of the illness.^7^ Such findings indicate that the dengue virus may have a more profound and lasting impact on cognitive function than previously understood. The potential for dengue to cause such significant neurological sequelae raises critical questions about the disease’s long-term effects on the brain and cognitive health.^8^

This systematic review assessed the existing research to better understand the association between dengue virus infection and cognitive impairments, such as memory loss, confusion, dementia, and Alzheimer disease. Focusing on filling the knowledge gap about the long-term neurological effects of dengue is particularly noteworthy, given the disease’s expanding geographical spread and impact on global public health and economies. The intention to aid in the development of clinical practices and public health strategies, as well as to inform the medical community and guide policymaking, is commendable. The potential of this research to improve patient outcomes and contribute to managing the global burden of dengue is well emphasized.

## METHODS

This systematic review followed the preferred reporting items for systematic reviews and meta-analyses (PRISMA) guidelines (Table S1).^9^ We utilized the Nested Knowledge software (Nested Knowledge, St. Paul, MN, USA) to carry out the systematic review. The study protocol was registered with PROSPERO (CRD42024500017).

### Inclusion Criteria

We included studies examining patients diagnosed with dengue, regardless of age, sex, or geographical location. Our primary outcome of interest is to find the association between cognitive impairments and dengue virus infection. Eligible study designs include RCTs, cohort studies, case-control studies, and observational studies. We considered studies published from inception to January 18, 2024. Initially, there was no language restriction, but for practicality, only articles published in English or available with English translations were reviewed. A detailed inclusion criteria was represented in Table S2.

### Database Search

A comprehensive literature search was conducted in PubMed, EMBASE, and Web of Science from inception to January 18, 2024. The search strategy utilized a combination of keywords and medical subject headings related to “dengue,” “cognitive impairment,” “dementia,” “Alzheimer’s,” “memory loss,” and “confusion.” No filters or restrictions were applied to the search to ensure comprehensive literature capture. A detailed search strategy is provided in Table S3.

### Screening of Articles

Two independent reviewers screened articles using Nested Knowledge software. They initially screened titles and abstracts to filter out irrelevant studies, subsequently conducting full-text reviews for potentially relevant articles. In cases of disagreement between the reviewers, a third independent reviewer intervened to resolve any discrepancies, ensuring a thorough and unbiased selection process for the systematic review.

### Data Extraction

Data extraction was independently carried out by 2 reviewers using a tagging function in Nested Knowledge. We collected data on study characteristics, including study design, country, patient demographics, types of cognitive impairments, total sample size, and prevalence rates of these impairments. For assessing associations, we recorded the total numbers in both dengue virus–infected and non-infected groups and the number of events, specifically the patients who developed cognitive impairment. We organized the tagged data and exported it to Microsoft Excel for data analysis. Any discrepancies in data extraction were resolved through discussion or by consulting a third reviewer.

### Quality Assessment

The methodological quality of the included studies was evaluated using the Newcastle-Ottawa Scale (NOS).^10^ This scale, designed for nonrandomized studies, rates studies based on group selection, comparability, and outcome determination, considering factors like cohort representativeness, exposure accuracy, confounding control, and outcome reporting. With a maximum of 9 points, the NOS score reflects the study’s quality.

## RESULTS

### Literature Search

**Figure 1.**
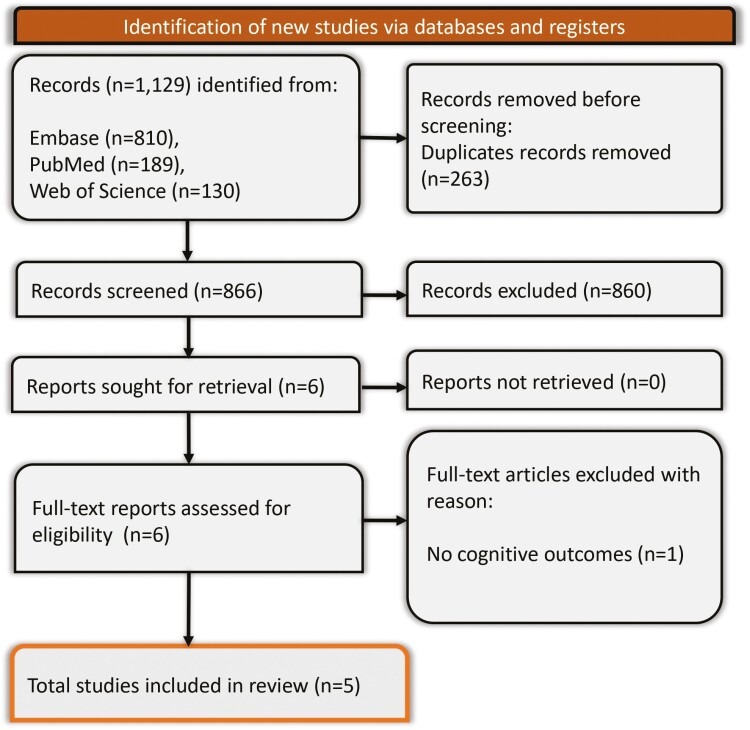
PRISMA flow diagram representing the screening and selection process of the studies.

The systematic search across multiple databases yielded a total of 1129 studies. After excluding 263 duplicates, 866 studies were screened based on titles and abstracts. Irrelevant studies were removed, including nonhuman studies, case reports, studies not related to dengue, reviews, short communications, and studies without cognitive outcomes. Six articles were retrieved for full-text screening, with 1 further excluded, for not reporting cognitive outcomes. Finally, 5 studies m*et al*l the eligibility criteria for inclusion in the systematic review, as shown in Figure 1.

### Characteristics of Included Studies

In 5 studies a total of 200 873 individuals, primarily over 45 years old, were involved from Taiwan, Brazil, and France (Table 1). These studies encompass a range of neurocognitive disorders, including Alzheimer disease, vascular dementia, unspecified dementia, memory loss, and confusion, examining their prevalence and associated risks in dengue patients. The participant data are divided into dengue (39 409) and nondengue (161 464) groups. The research, largely employing population-based cohort and prospective observational designs, reveals significant insights into the cognitive impact of dengue. Studies from Taiwan^8,11,12^ consistently report on dementia types in large cohorts, while studies from France^13^ and Brazil^14^ provide focused observations on cognitive symptoms in emergency and clinical settings. The studies rated a moderate quality on the NOS (Table S4).

**Table 1.** Summary characteristics of the studies included in the review

Study	Country	Study design	Age (mean)	Male %	Population	Total sample	Type of cognitive impairment	Patients infected with dengue	Cognitive impairmentzevents in infected	Nondengue patients	Cognitive impairment events in non-infected
^13^	France	Prospective cohort study	52	42.9	Patients tested positive for dengue in emergency departments	163	Confusion	37 (severe dengue), 126 (nonsevere dengue)	12 (severe dengue), 12 (nonsevere dengue)	NA	NA
^8^	Taiwan	Population-based cohort study	56	44.7	Patients aged 45+ years in National Health Insurance	1981	Unspecified dementia	389	23	1592	54
^11^	Taiwan	Population-based cohort study	60.71	46.3	Patients aged 45+ years diagnosed with dengue	189 640	Alzheimer disease	37 928	311	151 712	1046
							Vascular dementia	37 928	139	151 712	509
							Unspecified dementia	37 928	682	151 712	2447
^12^	Taiwan	Prospective observational study	59.66	47.9	Adults aged 45+ years diagnosed with dengue	8976	Alzheimer disease	816	5	8160	17
							Vascular dementia	816	4	8160	22
							Unspecified dementia	816	25	8160	101
^14^	Brazil	Prospective observational study	NA	37.2	Patients aged 14+ years with suspected dengue	113	Memory loss	113	18	NA	NA
							Reasoning problems	113	15	NA	NA

### Acute or Short-Term Cognitive Impairments

Only 2 studies have addressed short-term cognitive impairments associated with dengue, focusing primarily on confusion and memory loss. A prospective cohort study by^13^ in France, involving 163 patients diagnosed with dengue (126 with nonsevere and 37 with severe dengue) in the emergency department, reported 24 cases of confusion—12 in patients with nonsevere dengue and 12 in patients with severe dengue—with a significant association with severity (*P* =.001).^13^ Similarly, a study by^14^ in Brazil assessed 113 patients aged 14 years and older with suspected dengue and found significant short-term memory loss in 18 patients and reasoning problems in 15.^14^

### Long-Term Cognitive Impairments

Three studies have explored the association between dengue infection and long-term cognitive impairments. Chang et al.^8^ conducted a population-based cohort study in Taiwan with 1981 patients aged 45 years and older. Of 389 patients diagnosed with dengue, 23 developed unspecified dementia compared with 54 out of 1592 nondengue patients.^8^ Chien et al.^11^ analyzed 189 640 individuals, identifying 311 cases of Alzheimer disease, 139 cases of vascular dementia, and 682 cases of unspecified dementia among 37 928 dengue patients. In contrast, among 151 712 nondengue individuals, there were 1046 cases of Alzheimer disease, 509 cases of vascular dementia, and 2447 cases of unspecified dementia.^11^ Chu et al.,^12^ in another study with 8976 adults, reported 5 cases of Alzheimer disease, 4 cases of vascular dementia, and 25 cases of unspecified dementia among 816 dengue patients compared with 17, 22, and 101 cases, respectively, among 8160 nondengue patients.^12^

## DISCUSSION

To our knowledge, this is the first systematic review to provide significant insights into the potential association between dengue virus infection and cognitive impairments, a relatively underexplored domain in dengue-related research. Drawing on evidence from over 200 000 individuals, our findings indicate a notable prevalence of cognitive impairments among dengue patients and a higher risk compared with non-infected individuals. The results reveal dengue might cause acute cognitive dysfunctions, such as confusion, memory loss, and reasoning problems, which can impact patient care during the early stages of the disease. Studies also suggest a possible link between dengue and an increased risk of long-term cognitive impairments, including Alzheimer disease, vascular dementia, and unspecified dementia, particularly in older adults.

Previous studies on cognitive impairment in patients with dengue virus infection have shown that acute and short-term impairments, such as confusion, memory loss, and reasoning problems, significantly impact patient care during the early stages of the disease. These cognitive impairments are particularly in severe cases of dengue. Carras et al.^13^ identified that confusion was associated with severe dengue in dengue patients admitted to emergency departments. Teixeira et al.^14^ also reported memory loss and reasoning difficulties among younger patients, demonstrating the broad cognitive impacts that can occur soon after infection. These findings confirm that the dengue virus induces neurological effects beyond its traditional symptoms of fever and hemorrhagic manifestations.

Acute cognitive impairment usually appears during the viremic phase, within the first 4 to 7 days of illness, when the virus is actively replicating in the host.^15^ Clinical manifestations can range from mild confusion to severe neuropsychiatric conditions like encephalitis, seen in approximately 0.5% to 6% of hospitalized cases, and seizures, observed in about 0.1% to 2% of cases.^16^ These deficits result from the virus directly invading the CNS and from systemic inflammatory responses that disrupt brain function.^17^

DENV is believed to cross the blood-brain barrier (BBB) through infection of endothelial cells or a “Trojan horse” mechanism, where infected monocytes or dendritic cells act as vectors into the CNS.^18^ Once in the CNS, viral replication can lead to neuronal dysfunction primarily through mechanisms like oxidative stress and excitotoxicity.^19^ The excessive release of pro-inflammatory cytokines, including tumor necrosis factor-alpha, interleukin-6, and interferon-gamma, is central to neuroinflammation during dengue infection. Elevated levels of these cytokines have been reported in up to 70% of patients with severe dengue.^20^ This cytokine storm can activate microglia, the brain’s resident immune cells, which, when persistently activated, contribute to neuronal damage and cognitive impairment.^17^

In addition to viral neuroinvasion, systemic effects from severe dengue, particularly dengue hemorrhagic fever (DHF) and dengue shock syndrome (DSS), can further exacerbate cognitive impairment. Vascular leakage and shock in DHF and DSS can lead to hypoperfusion and hypoxia, contributing to transient cognitive disturbances.^16^ In severe cases, mortality rates in DHF and DSS can reach 10% to 20% without proper treatment.^21^ Patients with DHF and DSS are at significantly higher risk for neurological complications, with studies indicating that up to 12% of patients with severe dengue exhibit some form of cognitive impairment during acute infection.^20^ Hepatic dysfunction, present in 60% to 80% of patients with severe dengue, may lead to hepatic encephalopathy, further altering mental status and contributing to cognitive impairment.^22^

In the short term, these cognitive impairments are generally reversible as the acute infection resolves. Most patients show improvement in cognitive function as fever subsides and systemic inflammation decreases.^16^ Recovery times vary, but studies have reported cognitive recovery within 2 to 4 weeks following the acute phase for most patients.^20^ However, in a subset of patients, particularly those with severe or prolonged illness, cognitive deficits may persist for several months, likely due to residual inflammation or complications like dehydration, electrolyte imbalance, or secondary infections.^7^ Additionally, the lack of baseline cognitive assessments in older patients presents a limitation, as it becomes challenging to measure the true impact of dengue on cognitive function. Some older patients may have had preexisting cognitive impairments before infection, which could affect the accuracy of cognitive decline assessments following dengue.^11^

The pathophysiological processes that might contribute to these acute cognitive effects include neuroinflammation triggered by the viral infection, as indicated by the elevation of pro-inflammatory cytokines.^23^ This inflammation could disrupt normal neurological function, leading to the cognitive symptoms observed. Moreover, direct viral invasion of neural tissue, vascular hypo-perfusion, and metabolic disturbances during the acute phase of infection may exacerbate these effects, though these mechanisms require further elucidation through targeted biological studies.^24^

Regarding long-term outcomes, chronic cognitive impairments such as Alzheimer disease, vascular dementia, and unspecified dementia have been investigated. A study by^11^ proposed that while the incidence of dementia appeared elevated among dengue patients, the relationship might not be causal, as sensitivity analyses indicated the potential influence of unmeasured confounders.^11^ While acute cognitive impairment associated with dengue is well-documented, the long-term cognitive consequences remain less clearly defined.^25^ Emerging evidence suggests that dengue infection may lead to lasting cognitive effects, particularly in patients who experience severe neurological involvement during the acute phase.^26^ These long-term sequelae likely stem from different pathophysiological mechanisms than those responsible for acute cognitive changes, often involving chronic processes.^17^

One proposed mechanism for long-term cognitive decline is persistent low-grade inflammation that continues even after the resolution of the acute infection.^16^ Chronic neuroinflammation, even at subclinical levels, has been linked to cognitive decline in neurodegenerative diseases such as Alzheimer and Parkinson diseases.^20^ Although direct evidence for persistent neuroinflammation following dengue infection is limited, studies in other flavivirus infections, such as West Nile virus, suggest that chronic inflammation resulting from immune response remnants or viral particles could contribute to cognitive impairment.^27^ In severe dengue cases, elevated levels of interleukin-6 and tumor necrosis factor-alpha have been detected for up to 6 months post infection, which may explain ongoing neuroinflammation in some patients.^28^

Another contributing factor to long-term cognitive impairment could be ischemic brain damage resulting from repeated or prolonged episodes of hypo-perfusion during the acute phase.^17^ Ischemia-reperfusion injury, commonly observed in cases of dengue shock syndrome, can result in delayed neuronal death, leading to irreversible damage to critical brain structures like the hippocampus, which is essential for memory formation and retrieval.^22^ Studies have reported that 20% to 30% of patients with dengue-related encephalopathy exhibit evidence of hippocampal atrophy on neuroimaging, which may correlate with memory deficits observed in the months following recovery.^15^

BBB disruption during acute dengue infection may also play a role in long-term cognitive decline. During the acute phase, DENV can compromise the integrity of the BBB, leading to increased permeability. Although the BBB typically begins to repair itself after the acute infection resolves, chronic disruption has been reported in other viral infections and may allow peripheral immune cells and inflammatory mediators to continuously infiltrate the CNS.^17^ Neuroimaging studies in patients with a history of dengue encephalitis have shown white matter lesions in approximately 15% to 20% of cases, which may contribute to ongoing cognitive decline.^16^ In addition to these biological factors, psychological stressors related to the severe illness experience may also contribute to long-term cognitive outcomes.^29^ Studies have shown that up to 30% of patients recovering from severe dengue develop post-traumatic stress disorder, and 20%-40% experience depression, both of which have been associated with cognitive dysfunction.^30^ The prolonged recovery time and fear of recurrent infection may further exacerbate cognitive symptoms in some patients.^16^

Another important factor to consider is the potential for cognitive decline in dengue patients to be linked to the decompensation of preexisting cognitive impairments and other comorbidities.^31^ Literature suggests that patients with conditions such as diabetes and cardiovascular disease are more prone to decompensatory episodes during dengue infection, which can lead to complications like dizziness, increased fall risk, and cardiopulmonary instability, often necessitating emergency care.^32–34^ These episodes may exacerbate cognitive symptoms in affected patients, complicating the assessment of dengue’s direct impact on cognitive function. Recognizing the role of such comorbidities is essential for a more comprehensive understanding of cognitive outcomes in dengue patients.

The findings of this systematic review emphasize the need for increased clinical attention to cognitive outcomes in dengue patients, both during the acute phase and in the long term. While acute cognitive impairment tends to be reversible in most cases, clinicians should be vigilant in monitoring for potential long-term sequelae, particularly in patients with severe neurological involvement or multiple dengue infections. Early identification and intervention for cognitive impairment, including cognitive rehabilitation and pharmacological therapies such as anti-inflammatory agents, may help mitigate long-term deficits and improve patients’ quality of life.^35^

Further research is essential to elucidate the precise pathophysiological mechanisms underlying long-term cognitive decline following dengue infection.^16^ Longitudinal studies tracking cognitive function in dengue patients over time, coupled with advanced neuroimaging and biomarker analysis, are necessary to understand the chronic effects of dengue on the CNS.^17^ In particular, investigating the role of persistent inflammation, BBB integrity, and hippocampal damage in long-term cognitive outcomes will be critical for developing effective therapeutic interventions. Future research should also explore potential neuroprotective agents and anti-inflammatory treatments to prevent or reduce long-term cognitive impairment in dengue patients. While acute cognitive impairment in dengue patients is generally reversible, long-term cognitive sequelae may occur, especially in patients with severe infection. Chronic neuroinflammation, BBB disruption, ischemic injury, and psychological factors likely contribute to these outcomes. Understanding these processes is crucial for developing effective interventions to prevent and manage cognitive decline in dengue patients.^16,17^

While these insights are valuable, they come from studies with significant limitations, including small sample sizes and the retrospective nature of data collection, which may introduce biases such as misclassification, and incomplete follow-up.^12^ The heterogeneity in study designs and populations further limits the generalizability of the findings. Many studies have used health insurance databases, which can lead to inaccurate diagnoses and insufficient follow-up data. The lack of prospective, longitudinal studies weakens the ability to establish a causal relationship between dengue infection and cognitive decline, especially regarding long-term cognitive impairments. Additionally, the absence of research on younger populations and more diverse geographical regions restricts our understanding of how dengue-related cognitive impairments may vary across different demographic groups. These limitations emphasize the need for more rigorous, well-designed research to confirm the association between dengue and cognitive decline, while also highlighting the importance of ongoing monitoring and supportive care for affected patients.

Despite these limitations, this systematic review has several strengths. The review followed a comprehensive search strategy and adhered to PRISMA guidelines, ensuring methodological rigor. The use of robust tools like the NOS for quality assessment. The careful data extraction process and resolution of discrepancies by multiple reviewers further strengthened the reliability and accuracy of the results. However, the heterogeneity of the included studies, along with the exclusion of non-English articles, may have restricted the scope of the analysis. The reliance on observational data and the potential for publication bias limit the ability to establish causality.

Future research should prioritize study designs involving larger and more diverse populations, as well as exploring the biological mechanisms linking dengue to cognitive impairments. It is essential to clarify whether these cognitive effects result directly from the viral infection or are secondary to systemic effects. Currently, dengue treatment focuses on supportive care, managing fluid balance, and addressing complications. Prevention efforts emphasize mosquito control and bite avoidance alongside ongoing vaccine research, though no widely approved vaccine is available at present.

## CONCLUSION

This systematic review suggests that dengue virus infection may affect cognitive function in both acute and long-term contexts. However, the current evidence is not strong enough to establish a conclusive link. Further research with larger sample sizes and longitudinal studies is essential to confirm the impact of dengue virus on cognitive health.

## Supplementary Material

pyae053_suppl_Supplementary_Materials

## Data Availability

The data is with the authors and available on request.

## References

[1] Lindsey N , Lehman J, Staples J, Fischer M. Division of vector-borne diseases. national center for emerging and zo-onotic infectious diseases, CDC west Nile virus and other arboviral diseases—United States. CDC. 2013;63:521-526. PMC5779373 24941331

[2] Ilic I , Ilic M. Global patterns of trends in incidence and mortality of dengue, 1990-2019: an analysis based on the global burden of disease study. Medicina (Kaunas). 2024;60:425. 38541151 10.3390/medicina60030425 PMC10972128

[3] WHO Guidelines Approved by the Guidelines Review Committee. Dengue: Guidelines for Diagnosis, Treatment, Prevention and Control: New Edition. World Health Organization; 2009. 23762963

[4] Cam BV , Fonsmark L, Hue NB, Phuong NT, Poulsen A, Heegaard ED. Prospective case-control study of encephalopathy in children with dengue hemorrhagic fever. Am J Trop Med Hyg. 2001;65:848-851. 11791985 10.4269/ajtmh.2001.65.848

[5] Guzman MG , Gubler DJ, Izquierdo A, Martinez E, Halstead SB. Dengue infection. Nat Rev Dis Primers. 2016;2:16055. 27534439 10.1038/nrdp.2016.55

[6] Misra UK , Kalita J, Syam UK, Dhole TN. Neurological manifestations of dengue virus infection. J Neurol Sci. 2006;244:117-122. 16524594 10.1016/j.jns.2006.01.011

[7] Li G-H , Ning Z-J, Liu Y-M, Li X-H. Neurological manifestations of dengue infection. Front Cell Infect Microbiol. 2017;7:449. 29119088 10.3389/fcimb.2017.00449 PMC5660970

[8] Chang SH , Chang R, Su CS, et al Incidence of dementia after dengue fever: results of a longitudinal population-based study. Int J Clin Pract. 2021;75:e14318. 34180565 10.1111/ijcp.14318

[9] Page MJ , McKenzie JE, Bossuyt PM , et al The PRISMA 2020 statement: an updated guideline for reporting systematic reviews. Int J Surg. 2021;88:105906. 33789826 10.1016/j.ijsu.2021.105906

[10] Stang A. Critical evaluation of the Newcastle-Ottawa scale for the assessment of the quality of nonrandomized studies in meta-analyses. Eur J Epidemiol. 2010;25:603-605. 20652370 10.1007/s10654-010-9491-z

[11] Chien Y-W , Shih H-I, Wang Y-P, Chi C-Y. Re-examination of the risk of dementia after dengue virus infection: a population-based cohort study. PLoS NeglTrop Dis. 2023;17:e0011788. 10.1371/journal.pntd.0011788 PMC10699621 38055695

[12] Chu C-S , Tsai S-J, Cheng C-M, et al Dengue and dementia risk: a nationwide longitudinal study. J Infect. 2021;83:601-606. 34454958 10.1016/j.jinf.2021.08.037

[13] Carras M , Maillard O, Cousty J, et al Associated risk factors of severe dengue in Reunion Island: a prospective cohort study. PLoS NeglTrop Dis. 2023;17:e0011260. 10.1371/journal.pntd.0011260 PMC10138848 37068115

[14] Teixeira LAS , Nogueira FPS, Nascentes GAN. Prospective study of patients with persistent symptoms of dengue in Brazil. Rev Inst Med Trop São Paulo. 2017;59:e65. 28876417 10.1590/S1678-9946201759065 PMC5587034

[15] Trivedi S , Chakravarty A. Neurological complications of dengue fever. Curr Neurol Neurosci Rep. 2022;22:515-529. 35727463 10.1007/s11910-022-01213-7 PMC9210046

[16] Pinheiro JR , Camilo Dos Reis E, Souza R, et al Comparison of neutralizing dengue virus B cell epitopes and protective T cell epitopes with those in three main dengue virus vaccines. Front Immunol. 2021;12:715136. 34489965 10.3389/fimmu.2021.715136 PMC8417696

[17] Calderón-Peláez M-A , Velandia-Romero ML, Bastidas-Legarda LY, Beltrán EO, Camacho-Ortega SJ, Castellanos JE. Dengue virus infection of blood–brain barrier cells: consequences of severe disease. Front Microbiol. 2019;10:1435. 31293558 10.3389/fmicb.2019.01435 PMC6606788

[18] Yu J , Li X, Zhou D, et al Vimentin inhibits dengue virus type 2 invasion of the blood-brain barrier. Front Cell Infect Microbiol. 2022;12:868407. 35433510 10.3389/fcimb.2022.868407 PMC9005901

[19] Oliveira DS , Britto DG, de Sá GF, et al Blood components requirement in Brazilian dengue outbreaks: a retrospective analysis between 2008 to 2019. Hematol Transfus Cell Ther. 2024;46:381-386. 37690978 10.1016/j.htct.2023.07.006 PMC11451380

[20] Kalayanarooj S , Nimmannitya S. Is dengue severity related to nutritional status. Southeast Asian J Trop Med Public Health. 2005;36:378-384. 15916044

[21] Verma P , Baskey U, Choudhury KR, et al Changing pattern of circulating dengue serotypes in the endemic region: an alarming risk to the healthcare system during the pandemic. J Infect Public Health. 2023;16:2046-2057. 37944366 10.1016/j.jiph.2023.10.014

[22] Sami CA , Tasnim R, Hassan SS, et al Clinical profile and early severity predictors of dengue fever: current trends for the deadliest dengue infection in Bangladesh in 2022. IJID Reg. 2023;9:42-48. 37859805 10.1016/j.ijregi.2023.09.001 PMC10582778

[23] Tan S , Chen W, Kong G, Wei L, Xie Y. Peripheral inflammation and neurocognitive impairment: correlations, underlying mechanisms, and therapeutic implications. Front Aging Neurosci. 2023;15:1305790. 38094503 10.3389/fnagi.2023.1305790 PMC10716308

[24] Ortiz-Guerrero G , Gonzalez-Reyes RE, de-la-Torre A, Medina-Rincón G, Nava-Mesa MO. Pathophysiological mechanisms of cognitive impairment and neurodegeneration by toxoplasma Gondii Infection. Brain Sci. 2020;10:369. 32545619 10.3390/brainsci10060369 PMC7349234

[25] Belaunzarán-Zamudio PF , Ortega-Villa AM, Mimenza-Alvarado AJ, et al Comparison of the impact of Zika and dengue virus infection, and other acute illnesses of unidentified origin on cognitive functions in a prospective cohort in Chiapas Mexico. Front Neurol. 2021;12:631801. 33828518 10.3389/fneur.2021.631801 PMC8019918

[26] Patel JP , Saiyed F, Hardaswani D. Dengue fever accompanied by neurological manifestations: challenges and treatment. Cureus. 2024;16:e60961. 38910682 10.7759/cureus.60961 PMC11193856

[27] Lin H-C , Chou H-P, Chiang Y-C, Chang R, Chen Y-S, Juan Y-C. Neurological or psychiatric disorders after dengue fever. JAMA Netw Open. 2024;7:e2410075. 38713469 10.1001/jamanetworkopen.2024.10075 PMC11077384

[28] Samaan Z , McDermid Vaz S, Bawor M, Potter TH, Eskandarian S, Loeb M. Neuropsychological impact of west nile virus infection: an extensive neuropsychiatric assessment of 49 cases in Canada. PLoS One. 2016;11:e0158364. 27352145 10.1371/journal.pone.0158364 PMC4924871

[29] Tizenberg BN , Brenner LA, Lowry CA, et al Biological and psychological factors determining neuropsychiatric outcomes in COVID-19. Curr Psychiatry Rep. 2021;23:1-25. 10.1007/s11920-021-01275-3 PMC8485771 34648081

[30] Dinakaran D , Sreeraj VS, Venkatasubramanian G. Dengue and psychiatry: manifestations, mechanisms, and management options. Indian J Psychol Med. 2022;44:429-435. 36157026 10.1177/02537176211022571 PMC9460008

[31] Tsai J-J , Chokephaibulkit K, Chen P-C, et al Role of cognitive parameters in dengue hemorrhagic fever and dengue shock syndrome. J Biomed Sci. 2013;20:88. 24305068 10.1186/1423-0127-20-88 PMC4174897

[32] Halstead S , Wilder-Smith A. Severe dengue in travellers: pathogenesis, risk and clinical management. J Travel Med. 2019;26:taz062. 31423536 10.1093/jtm/taz062

[33] Lu HZ , Xie YZ, Gao C, et al Diabetes mellitus as a risk factor for severe dengue fever and West Nile fever: a meta-analysis. PLoS NeglTrop Dis. 2024;18:e0012217. 10.1371/journal.pntd.0012217 PMC11168630 38820529

[34] Tejo AM , Hamasaki DT, Menezes LM, Ho Y-L. Severe dengue in the intensive care unit. J Intensive Med. 2024;4:16-33. 38263966 10.1016/j.jointm.2023.07.007 PMC10800775

[35] Haller OJ , Semendric I, George RP, Collins-Praino LE, Whittaker AL. The effectiveness of anti-inflammatory agents in reducing chemotherapy-induced cognitive impairment in preclinical models–a systematic review. Neurosci Biobehav Rev. 2023;148:105120. 36906244 10.1016/j.neubiorev.2023.105120

